# Design and Thermodynamic Analysis of a Novel Solar CBS-PVT System Using Film-Based Beam Splitting Technology

**DOI:** 10.3390/e26010031

**Published:** 2023-12-27

**Authors:** Gang Wang, Jialin Liu, Zeshao Chen

**Affiliations:** 1 School of Energy and Power Engineering, Northeast Electric Power University, Jilin 132012, China; kinggang@neepu.edu.cn (G.W.); judevisalli868@gmail.com (J.L.); 2 School of Engineering Science, University of Science and Technology of China, Hefei 230027, China

**Keywords:** CBS-PVT, solar energy, spectral filter, HTC, hybrid PV/thermal

## Abstract

An innovative solar concentrating beam splitting photovoltaic thermal (CBS-PVT) system using a half-trough concentrator (HTC) and a film spectrum filter (FSF) is proposed and studied in this study. The FSF used for this system is designed and its average reflectivity and transmissivity are 0.272 and 0.728 for the full spectrum range. Performance evaluation results of the CBS-PVT system reveal the design correctness of the system. When the N-S solar tracking error (STE) rises to 0.15°, the optical efficiency of the entire CSB-PVT system can be kept at 0.8653, showing good adaptable capacity to the STE. The operation feasibility analysis shows that the PV efficiency of the PV subsystem (PVS) is 0.314 and the overall system efficiency overall is 0.26. Parametric analysis results indicate that when the solar thermal collector tube (TCT) operating temperature rises, the total power and overall efficiency of the CSB-PVT system both rise first and then decrease. When the TCT temperature is about 225 °C, the CBS-PVT system reaches its maximum output power of 1003.6 W and the maximum overall efficiency of 0.261. When the PV cell module (PVCM) temperature increases, the total power and overall efficiency of the CBS-PVT system decrease linearly. When the PVCM temperature rises to 50 °C, the two parameters decrease to 952.9 W and 0.248.

## 1. Introduction

Solar energy is clean and inexhaustible, and has been widely studied in recent years [1,2,3,4]. Solar thermal, PV power, solar photochemistry, and solar photobiological utilizations are several main solar application approaches. In the current state, solar thermal [5,6,7] and PV power [8,9,10] are the main ways to achieve large-scale solar utilizations.

In PV power generation, to reduce the solar power costs, it will be effective to increase the solar intensity by using solar concentration to reduce the number of PV cells. Among different kinds of solar concentrators, the parabolic trough concentrator (PTC) is the most mature in the world and can be applied in both solar PV and thermal power systems. In addition, the operating characteristics of PV cells depends on the solar spectrum. Solar PV–thermal systems using spectral splitting can solve this problem to a certain degree [11,12,13,14]. It is a cutting-edge technology in the field of solar energy utilization research. Generally, the spectral filter (SF) for PV–thermal systems can be divided into a solid film spectral filter (FSF) and a nanofluid spectral filter (NSF) [15]. When the PV–thermal system employs the SF and the solar concentrator at the same time, the technology can be called concentrating beam splitting (CBS) technology. The CBS technology can be utilized in PV–thermal systems to increase the overall system efficiencies. In contrast with the traditional PV–thermal technology, the CBS technology can make the output heat transfer fluid temperature of the thermal utilization not limited by the PV cell temperature, thus improving the grade of output thermal energy as well as extending the application (especially the thermal utilization) scenarios of the PV–thermal system.

Many studies on different types of PV–T systems have been reported. Jiang et al. [16] proposed a two-stage concentrated PV–thermal hybrid system using PTC. A dimensionless optical model with the focal length of the PTC as the characteristic length was established. The SF was designed and its optical performance was tested. Liu et al. [17] proposed a new PV–thermal system with LFR and FSF. A performance analysis model of medium- and high-temperature solar energy utilization system was established and the total efficiency was calculated. They found that the PV–thermal system had higher efficiency compared with the pure concentrating PV (C-PV) system, and the overall efficiency of the system was less affected by temperature in contrast with the C-PV system. Todd et al. [18] studied the PV–thermal system with NSF and PTC. The nanofluids consisted of PDMS, ITO, and Au nanoparticles. The study indicated that the system electrical efficiency and thermal efficiency were 4% and 61%. A PV–thermal system based on a multimirror solar concentrator was designed by Wang et al. [19]. The thermodynamic estimation and optical evaluation of the PV–thermal system were conducted. Joshi and Dhoble [20] extensively classified and reviewed the research on PV–thermal systems in recent years, and mainly introduced the experimental works on different types of PV–thermal systems in the past decade.

The literature review reveals that for the practical applications of solar PV–thermal systems, large solar concentrators with high concentration ratio (*CR*) will bring a series of problems, for instance, high construction costs, high requirements for sun-tracking accuracy, the swing problem of solar receiver and photovoltaic cells with high installation height, etc. By contrast, solar concentrators with relatively lower *CR* will have better application potential for PV–thermal systems. Considering the maturity of PTC, the half-trough concentrator will be a good potential of a solar concentrator with low *CR*.

The main innovation of this study is the design and performance estimation of an innovative concentrating beam splitting photovoltaic thermal (CBS-PVT) system with a half-trough concentrator (HTC) and FSF. The CBS-PVT system and FSF are both designed. In addition, the working principle of the CBS-PVT system is given. The optical characteristics and thermodynamic estimations of the CBS-PVT system are carried out to reveal the design correctness and technical feasibility of the system. A parametric study is also launched to show the influences of typical factors on the operating performance of the CBS-PVT system.

## 2. Methods and Materials

### 2.1. Overall CBS-PVT System Design

**Figure 1:**
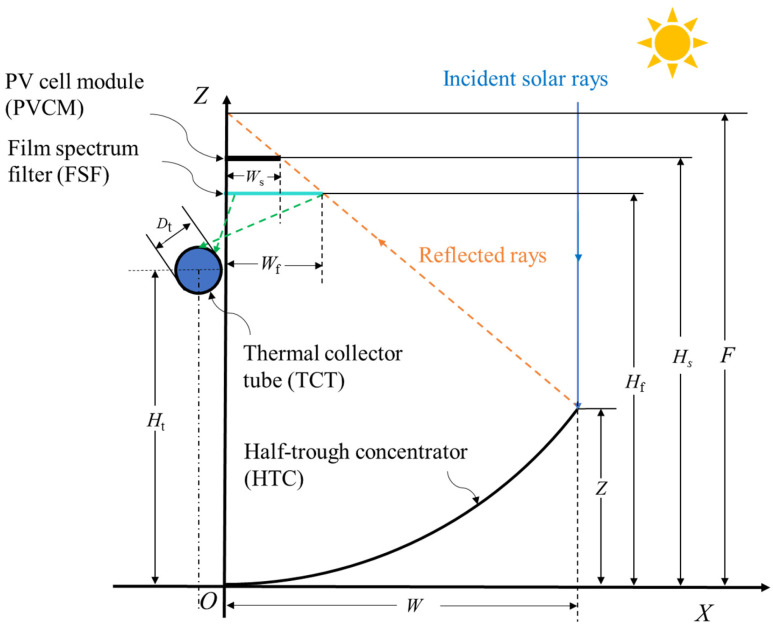
The CBS-PVT system diagram.

Figure 1 shows a diagram of the overall CBS-PVT system. The system is mainly composed of the HTC, FSF, PV cell module (PVCM), thermal collector tube (TCT), and necessary auxiliary structures. The PVCM is arranged above the FSF. When the incident sunlight is reflected by the HTC, part of the light passes through the FSF and reaches the surface of the PVCM to generate power. The other part of the light will be reflected to the TCT to heat the heat transfer fluid (HTF) such as water or heat transfer oil flowing in the TCT for thermal application. The heated water can be used in the low-temperature application field, and the heated heat transfer oil can be applied in both low- and middle-temperature utilization conditions. Based on the initial design principle, the CBS-PVT system cannot be designed to have a very large concentration ratio, as the large concentration ratio will bring the problems of high requirements for sun-tracking accuracy and the swing problem of solar receiver and PV cells with high installation height.

Figure 1 also provides the CBS-PVT system design principle. In Figure 1, for the HTC, the focal length is *F*, the depth is *Z*, and the width is *W*. The width and height of the FSF are *W*_f_ and *H*_f_. For the PVCM, its width is *W*_s_, and its height is *H*_s_. For the TCT, its diameter and height are *D*_t_ and *H*_t_.

The equation of the HTC is

(1) $$X^{2}=4FZ$$

The *CR* of the HTC is

(2) $$R_{g}=\frac{W-W_{s}}{W_{s}}$$

The relative aperture of the HTC is

(3) $$R_{a}=\frac{W}{F}$$

To study the operating characteristics of this CBS-PVT system, the geometric parameters were assumed and calculated, and are shown in Table 1.

### 2.2. Design of the FSF

The FSF used in this study is a spectrally selective transmission film. This study uses the Needle method to design and optimize the film system of the FSF for the CBS-PVT system. For monocrystalline silicon cells, the ideal FSF should have the following performances:

(4) $$\rho \left(\lambda \right)=\left\{\begin{array}{l} 0\;\;\;(380\;\mathrm{nm}\leq \lambda \leq 1100\;\mathrm{nm})\\ 1\;\;\;(\lambda <380\;\mathrm{nm},\lambda >1100\;\mathrm{nm}) \end{array}\right.$$

(5) $$\tau \left(\lambda \right)=\left\{\begin{array}{l} 1\;\;\;(380\;\mathrm{nm}\leq \lambda \leq 1100\;\mathrm{nm})\\ 0\;\;\;(\lambda <380\;\mathrm{nm},\lambda >1100\;\mathrm{nm}) \end{array}\right.$$

where $\rho \left(\lambda \right)$ and $\tau \left(\lambda \right)$ are the spectral reflectivity and spectral transmissivity of the FSF. λ is the wavelength of sunlight. For the spectral region of $\lambda_{1}\sim \lambda_{2}$ , the average reflectivity and average transmissivity of the FSF are defined as follows:

(6) $$\rho_{\mathrm{ave}}=\int_{\lambda_{1}}^{\lambda_{2}}\rho \left(\lambda \right)E\left(\lambda \right)d\lambda /\int_{\lambda_{1}}^{\lambda_{2}}E\left(\lambda \right)d\lambda$$

(7) $$\tau_{\mathrm{ave}}=\int_{\lambda_{1}}^{\lambda_{2}}\tau \left(\lambda \right)E\left(\lambda \right)d\lambda /\int_{\lambda_{1}}^{\lambda_{2}}E\left(\lambda \right)d\lambda$$

where $E\left(\lambda \right)$ is the spectral irradiance.

**Figure 2:**
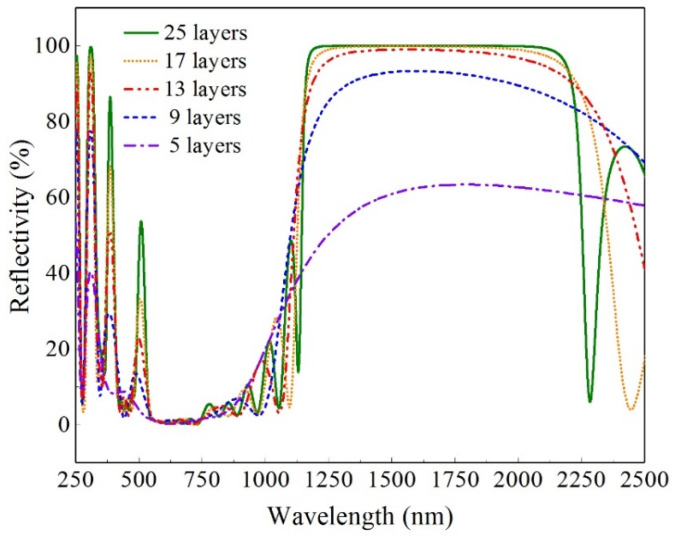
Spectral reflectivity of five designed FSFs.

For the FSF design, the three materials are niobium trioxide, sodium hexafluoroaluminate, and germanium. The TFCalc (V330) software is used to carry out the FSF design. It is a mature optical film design software based on the Needle method and has been used in many film design studies by relevant researchers [21,22]. The design results of the spectral reflectivity of several FSFs with different film layers are provided in Figure 2, and are calculated and obtained by using the TFCalc software. The spectral filtering characteristics of the FSFs are given in Table 2. According to Table 2, FSFs with five and nine film layers have smaller $\rho_{\mathrm{ave}}$ in 380~1100 nm, but higher $\tau_{\mathrm{ave}}$ in other wavelength ranges. The spectral filtering characteristics of the other three FSFs are similar. However, the FSF with 25 film layers can have the lowest $\tau_{\mathrm{ave}}$ in the wavelength range of <380 nm and >1100 nm compared to the other four FSFs. Therefore, the FSF with 25 film layers is selected to be used in the CBS-PVT system.

**Figure 3:**
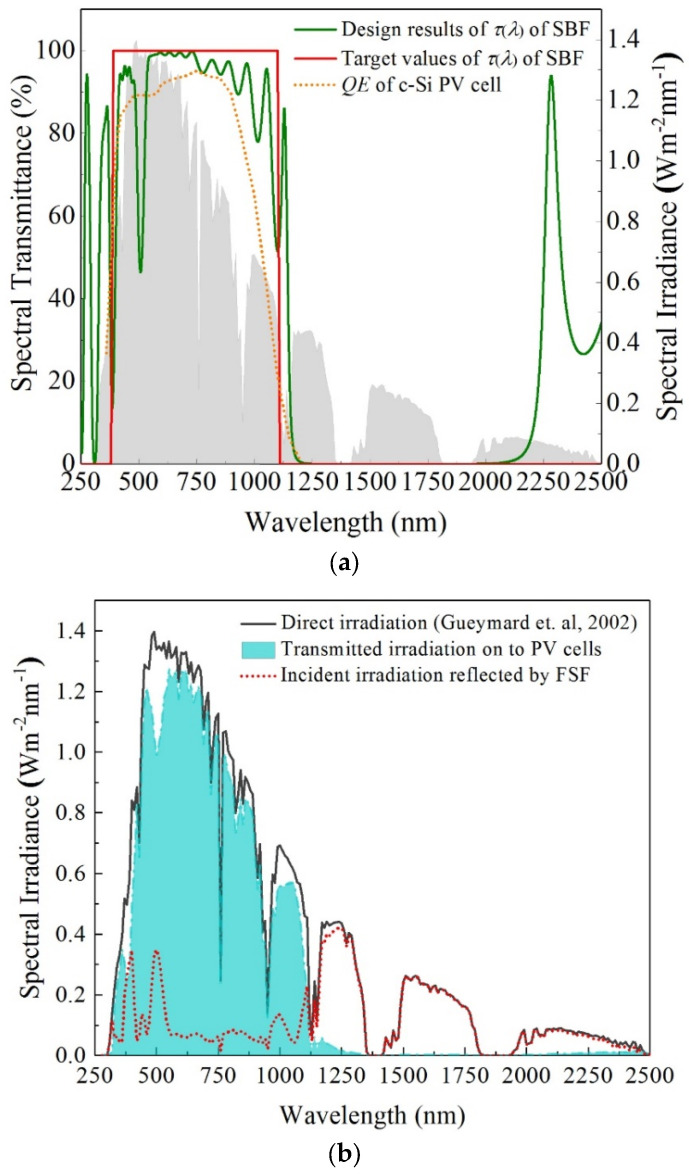
Spectral transmittance curves (**a**) and spectral filtering characteristics [24] (**b**) of the selected FSF.

Figure 3a presents the spectral transmissivity curves of the selected FSF as well as the QE curve of monocrystalline silicon cells. The spectral transmissivity curve of the selected FSF is obtained by using the TFCalc (V330) software, as mentioned above. The target value curve of spectral transmissivity of FSF is determined according to the spectral response range of monocrystalline silicon cells, which can be seen in Ref. [23]. The QE curve of monocrystalline silicon cells and the solar spectral irradiance data were initially obtained in early relevant experiments and can be found in Refs. [23,24]. Figure 3b illustrates the spectral irradiance conditions of incident light on the PVCM and TCT under the influence of the FSF. The results show that for 250~2500 nm, the average transmissivity and average reflectivity of the FSF are 0.728 and 0.272, respectively.

### 2.3. Modeling Approaches

For this study, the optical characteristics estimation of the CBS-PVT system are conducted by means of MCRT [25], which is a probabilistic and statistical method for randomly sampling a large number of incident rays. The optical simulation code used in this paper is TracePro software. The thermodynamic estimation of the CBS-PVT system should consider two subsystems, which are the photovoltaic subsystem (PVS) and heat utilization subsystem (HUS). The corresponding calculation formulas for the PVS (i.e., the PVCM) of the CBS-PVT system are [17] as follows:

(8) $$V_{\mathrm{oc},c,\mathrm{bs}}=hcV_{\mathrm{oc}}/\left(\lambda_{2}E_{g}\right)+n_{f}k_{B}T_{\mathrm{pvcm}}\ln\left(CR\right)/e$$

(9) $$I_{\mathrm{sc},c,\mathrm{bs}}=CR\cdot I_{\mathrm{sc},\mathrm{bs}}$$

(10) $$I_{\mathrm{sc},\mathrm{bs}}=\int_{0}^{\infty }\rho_{\mathrm{htc}}\left(\lambda \right)\cdot \tau_{\mathrm{fsf}}\left(\lambda \right)\cdot E_{s}\left(\lambda \right)\cdot QE\left(\lambda \right)\cdot \frac{e\lambda }{hc}\cdot A_{\mathrm{pvcm}}d\lambda$$

(11) $$Q_{\mathrm{pvcm},\mathrm{bs}}=Q_{\mathrm{htc},\mathrm{in}}\int_{0}^{\infty }\rho_{\mathrm{htc}}\left(\lambda \right)\tau_{\mathrm{fsf}}\left(\lambda \right)d\lambda$$

(12) $$P_{\mathrm{pvcm}}=FF\cdot V_{\mathrm{oc},c,\mathrm{bs}}\cdot I_{\mathrm{sc},c,\mathrm{bs}}$$

(13) $$\eta_{\mathrm{pvcm}}=P_{\mathrm{pvcm}}/Q_{\mathrm{pvcm},\mathrm{bs}}$$

where $P_{\mathrm{pvcm}}$ and $\eta_{\mathrm{pvcm}}$ are the maximum power and photovoltaic efficiency of the PVCM. *T*_pvcm_ and *A*_pvcm_ are the temperature and area of the PVCM. *ρ*_htc_ and *τ*_fsf_ are the spectral reflectivity of the HTC and spectral transmissivity of the FSF.

This study assumes that the solar heat absorbed by the TCT will be applied for generating power. The energy reflected to the TCT is [17] as follows:

(14) $$Q_{\mathrm{tct}}=Q_{\mathrm{htc},\mathrm{in}}\int_{0}^{\infty }\rho_{\mathrm{htc}}\left(\lambda \right)\rho_{\mathrm{fsf}}\left(\lambda \right)d\lambda$$

where *ρ*_fsf_ is the spectral reflectivity of the FSF.

If the convection heat loss of the TCT is neglected, the relevant formulas for the HUS of the CBS-PVT system are [17] the following:

(15) $$Q_{\mathrm{tct},a}=Q_{\mathrm{htc},\mathrm{in}}\int_{0}^{\infty }\tau_{\mathrm{tct},\mathrm{shell}}\left(\lambda \right)\rho_{\mathrm{htc}}\left(\lambda \right)\rho_{\mathrm{fsf}}\left(\lambda \right)\alpha_{\mathrm{tct}}\left(\lambda \right)d\lambda$$

(16) $$Q_{\mathrm{tct},\mathrm{loss}}=A_{\mathrm{tct}}\cdot \varepsilon \cdot \sigma \cdot (T_{\mathrm{tct}}^{4}-{T_{0}}^{4})$$

(17) $$\eta_{\mathrm{th}}=\frac{Q_{\mathrm{tct},\mathrm{net}}}{Q_{\mathrm{tct}}}=\frac{Q_{\mathrm{tct},a}-Q_{\mathrm{tct},\mathrm{loss}}}{Q_{\mathrm{tct}}}$$

where *T*_tct_ is the operation temperature of the TCT. *Q*_tct,a_ and *Q*_tct,loss_ represent the radiation energy absorbed by the TCT and the radiation loss of the TCT. *α*_tct_ is the spectral absorptivity of the TCT, *A*_tct_ is the surface area of the TCT, and *τ*_tct,shell_ is the spectrum transmissivity of the TCT shell.

The thermal, exergic, and PV efficiencies of the whole CBS-PVT system are as follows [17,26]:

(18) $$\eta_{\mathrm{sys},\mathrm{th}}=\frac{Q_{\mathrm{tct},\mathrm{net}}}{Q_{\mathrm{htc},\mathrm{in}}}=\frac{Q_{\mathrm{tct},a}-Q_{\mathrm{tct},\mathrm{loss}}}{Q_{\mathrm{htc},\mathrm{in}}}$$

(19) $$\eta_{\mathrm{sys},\mathrm{pv}}=\frac{P_{\mathrm{pvcm}}}{Q_{\mathrm{htc},\mathrm{in}}}$$

(20) $$\eta_{\mathrm{exe}}=\eta_{\mathrm{sys},\mathrm{th}}\left(1-\frac{T_{0}}{T_{\mathrm{tct}}}\right)+\eta_{\mathrm{sys},\mathrm{pv}}$$

where $T_{0}$ is the environment temperature and $\left(1-T_{0}/T_{\mathrm{tct}}\right)$ is the Carnot efficiency. According to the relevant literature [26], the efficiency of actual heat engine is about 2/3 of the Carnot efficiency. Hence, the overall efficiency of the CBS-PVT system is [26]

(21) $$\eta_{\mathrm{cbs}-\mathrm{pvt}}=\eta_{\mathrm{sys},\mathrm{pv}}+\frac{2}{3}\eta_{\mathrm{plant}}\cdot \eta_{\mathrm{pb},\mathrm{net}}\cdot \eta_{\mathrm{sys},\mathrm{th}}\left(1-\frac{T_{0}}{T_{\mathrm{tct}}}\right)$$

The electric power contributed by the HUS and that of the CBS-PVT system shall be [17,26]

(22) $$P_{\mathrm{tct}}=\frac{2}{3}\eta_{\mathrm{plant}}\cdot \eta_{\mathrm{pb},\mathrm{net}}\cdot Q_{\mathrm{tct},\mathrm{net}}\left(1-\frac{T_{0}}{T_{\mathrm{tct}}}\right)$$

(23) $$P_{\mathrm{total}}=P_{\mathrm{pvcm}}+P_{\mathrm{tct}}$$

## 3. Results and Discussions

### 3.1. Optical Characteristics

To estimate the optical characteristics of the CBS-PVT system, a numerical model was established and its structural parameters could be seen in Section 2.1. The concentration ratio of this HTC is 25.67, which is obtained by using the concentration ratio definition formula (i.e., Equation (2)) when the width of the HTC and the width of the PVCM are assumed. The height of the FSF is 1506.25 mm, and that of the PVCM is 1543.75 mm. The HTC is defined as a perfect mirror. As mentioned above, the FSF with 25 film layers is selected, and the average transmissivity and average reflectivity of the FSF are 0.728 and 0.272, which are calculated and obtained by using Equations (6) and (7). The solar energy flux density (EFD) is 990 W/m^2^. In this part of the current study, the concentrating process and the adaptable capacity to solar tracking error (*ε*_tr_) of the CBS-PVT system are analyzed.

**Figure 4:**
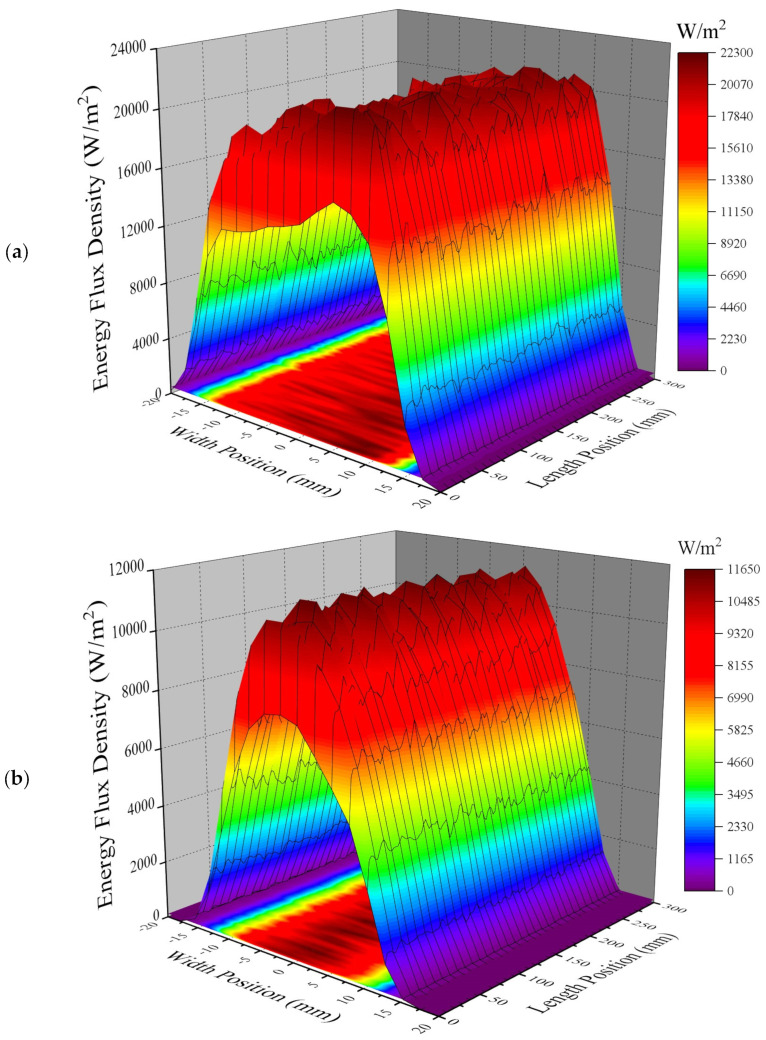
EFD maps on the PVCM (**a**) and on the TCT (**b**).

In Figure 4, the two three-dimensional color maps are the EFD distributions on the PVCM and on the TCT, respectively. Figure 4 demonstrates that the EFD curve on the PVCM shows a three-section slope shape and that on the TCT is unimodal. According to the calculation, the average EFDs on the PVCM and on the TCT are 18,464 W/m^2^ and 3448.2 W/m^2^. The total radiation flux on the PVCM is 166.2 W, and that reflected onto the TCT is 62.1 W. As a result, the optical efficiencies of the PVCM, TCT, and CBS-PVT system are all 0.988, which are very close.

**Figure 5:**
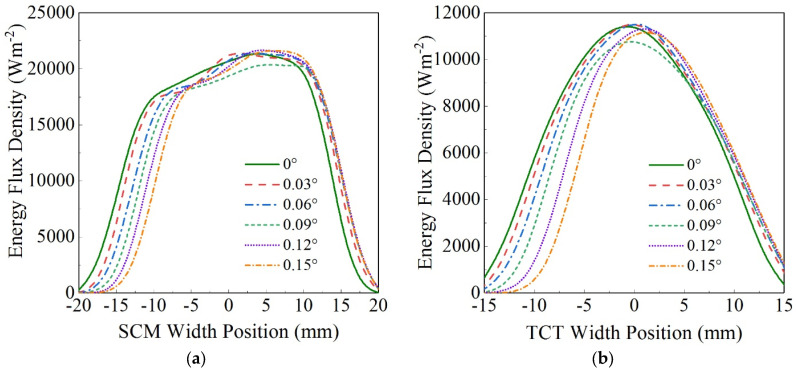
Effects of STE on EFD distributions on the PVCM (**a**) and on the TCT (**b**).

In order to study the adaptable capacity to STE of the CBS-PVT system, the MCRT method is used to simulate the optical process of the system under different N-S STE values. Figure 5 shows the EFD distributions on the PVCM and on the TCT when the STE changes. The results show that when the STE rises from 0° to 0.15°, the EFD curves on the PVCM and on the TCT both move to the right, but the EFD curve shape variations on the PVCM and on the TCT are relatively small. With the STE changes from 0° to 0.15°, the radiation energy on the TCT decreases from 62.1 W to 51.5 W, and that on the PVCM decreases from 166.2 W to 148.8 W.

**Figure 6:**
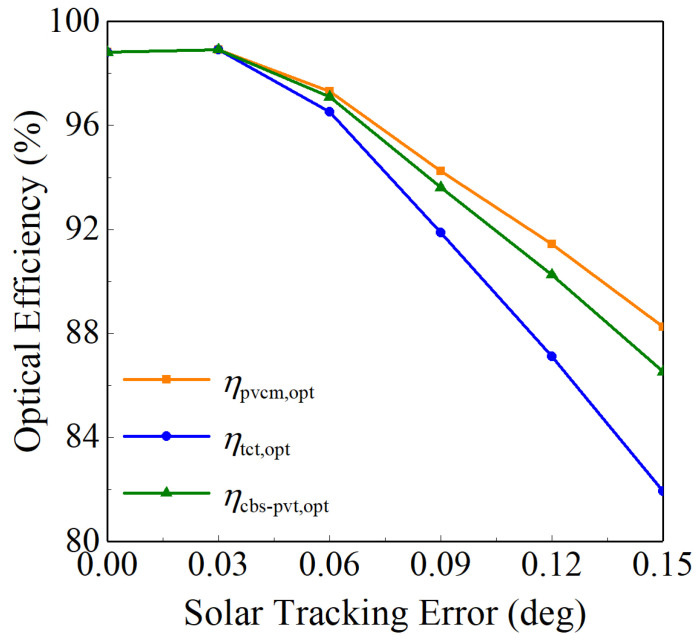
*η*_pvcm,opt_, *η*_tct,opt_, and *η*_cbs-pvt,opt_ variations when the STE changes.

The influences of STE on different optical efficiencies are shown in Figure 6. The results show that when the STE rises to 0.15°, the optical efficiency of the PVCM is $\eta_{\mathrm{pvcm},\mathrm{opt}}$ = 0.883, that of the TCT is $\eta_{\mathrm{tct},\mathrm{opt}}$ = 0.819, and that of the CBS-PVT system is $\eta_{\mathrm{cbs}-\mathrm{pvt},\mathrm{opt}}$ = 0.865.

### 3.2. Thermodynamic Estimation Results

The $\rho_{\mathrm{ave}}$ and $\tau_{\mathrm{ave}}$ of the FSF were given in Section 2.2. The solar cells used in this study are assumed to be monocrystalline silicon cells. Table 3 provides the relevant constants needed in the operation estimation for the CBS-PVT system. The solar intensity is 1000 W/m^2^. It is assumed that the length of the HTC is 5 m, so the effective solar receiving area of the HTC is 3.85 m^2^, and the incident energy *Q*_htc,in_ is 3850 W. For the PVCM, $V_{\mathrm{oc}}$ and *FF* are 0.706 V and 0.852. When *T*_pvcm_ is 30 °C, *n_f_* of the PVCM is 1.28, and *r*_s_ is 0.012.

The operating temperatures of the TCT and $T_{0}$ are 200 °C and 25 °C. The diameter of the TCT is 30 mm. The average absorptivity of the TCT and average transmission rate of the TCT shell are 0.95. In this section, only the radiant heat loss of the TCT is considered. When all parameters are settled, the operation estimation results of the CBS-PVT system are shown in Table 4. The output power of the PVS is 837.4 W, and that of the HUS is 162.1 W. The photoelectric efficiency of the PVS is 0.314 and the overall system efficiency *η*_cbs-pvt_ is 0.26.

Table 5 presents a comparison of the CBS-PVT system and some other PV–thermal systems reported by relevant studies [14,18,29]. As different SFs have different beam splitting performances, the overall electric efficiency and thermal efficiency values of different PV–thermal systems are also different. Compared with the other typical PV–thermal systems using NSFs presented in Table 5, the CBS-PVT system of this study can have simpler layout and much lower initial investment costs. That is because the use of FSF can simplify the SF structure and reduce the investment costs of the SF to a certain degree. For instance, compared with a PVT system unit using indium tin oxide–ethylene glycol (ITO-EG) nanofluids and HTC, the initial investment cost of the proposed CBS-PVT system unit discussed in this section can be reduced by about USD 357.6 when the two PVT system units have the same output power. In addition, compared with PV–thermal systems using parabolic trough or other large solar concentrators, the CBS-PVT system can have smaller field requirements as it employs the HTC. These points make the CBS-PVT system suitable for applications in both large-scale and distributed PV–thermal utilization conditions. The scale of CBS-PVT units can be designed according to the actual demand. If the application condition is the distributed power and thermal supply in rural areas, the CBS-PVT unit can be made relatively smaller. Under that condition, the CBS-PVT unit can operate separately, or two units operate together. If the application condition is the large-scale power and thermal supply in a large suburban industrial site, there may be many CBS-PVT units connected in multiple rows and columns, and the CBS-PVT unit size may be larger.

**Figure 7:**
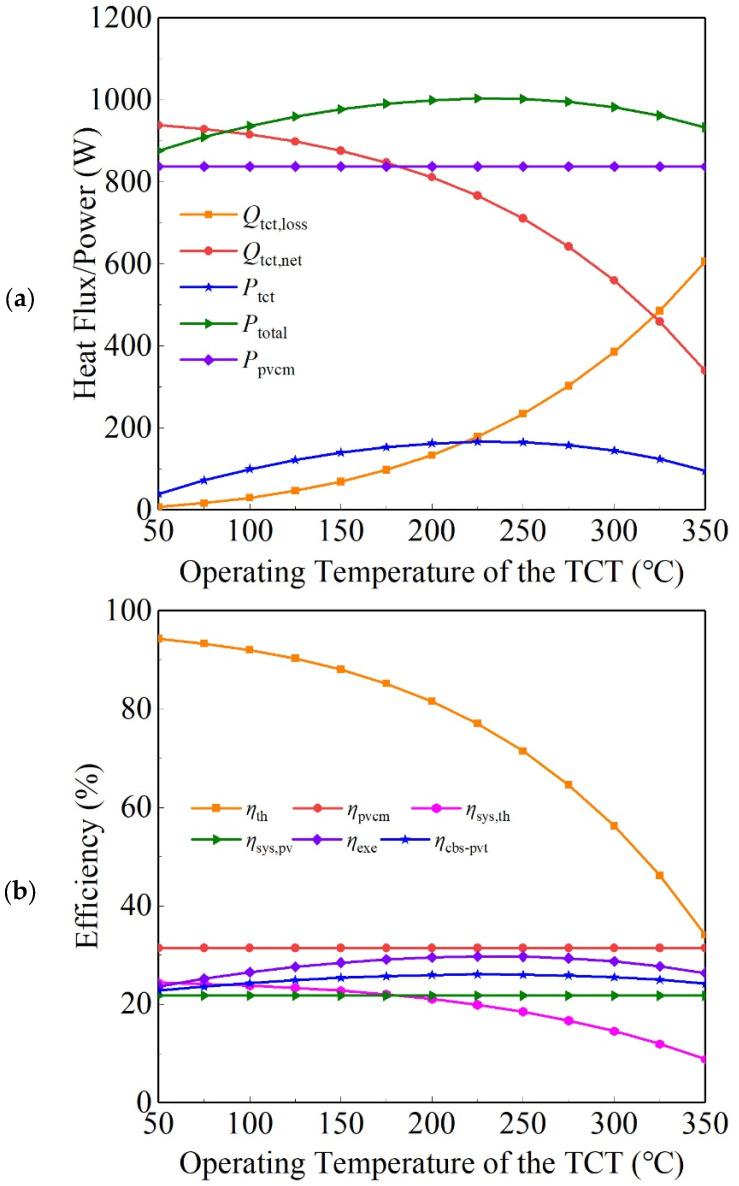
Powers (**a**) and efficiencies (**b**) of the CBS-PVT system when the TCT temperature changes.

In this section, the influences of two typical temperatures on the operating performance of the CBS-PVT system are estimated, which are the PVCM temperature *T*_pvcm_ and TCT operating temperature *T*_tct_. When *T*_pvcm_ is 30 °C and the solar irradiance is 1000 W/m^2^, with *T*_tct_ changed, the power and efficiency parameters of the CBS-PVT system are shown in Figure 7. As shown in Figure 7, when *T*_tct_ rises from 50 °C to 350 °C, the radiation energy loss *Q*_tct,loss_ of the TCT increases continuously; thus, the net heat flux *Q*_tct,net_ obtained by the TCT decreases. The output power *P*_th_ of the HUS and the total output power *P*_total_ have the same changing trend, and they both reach the maximum points (166.2 W and 1003.6 W) when *T*_tct_ is about 225 °C.

The increase in *Q*_tct,loss_ of the TCT leads to the reduction in *η*_th_ of the TCT as well as *η*_sys_,_th_ of the CBS-PVT system. When *T*_tct_ rises from 50 °C to 350 °C, *η*_exe_ and *η*_cbs-pvt_ of the CBS-PVT system both rise firstly and then drop down, and their maximal values are 0.297 and 0.261.

**Figure 8:**
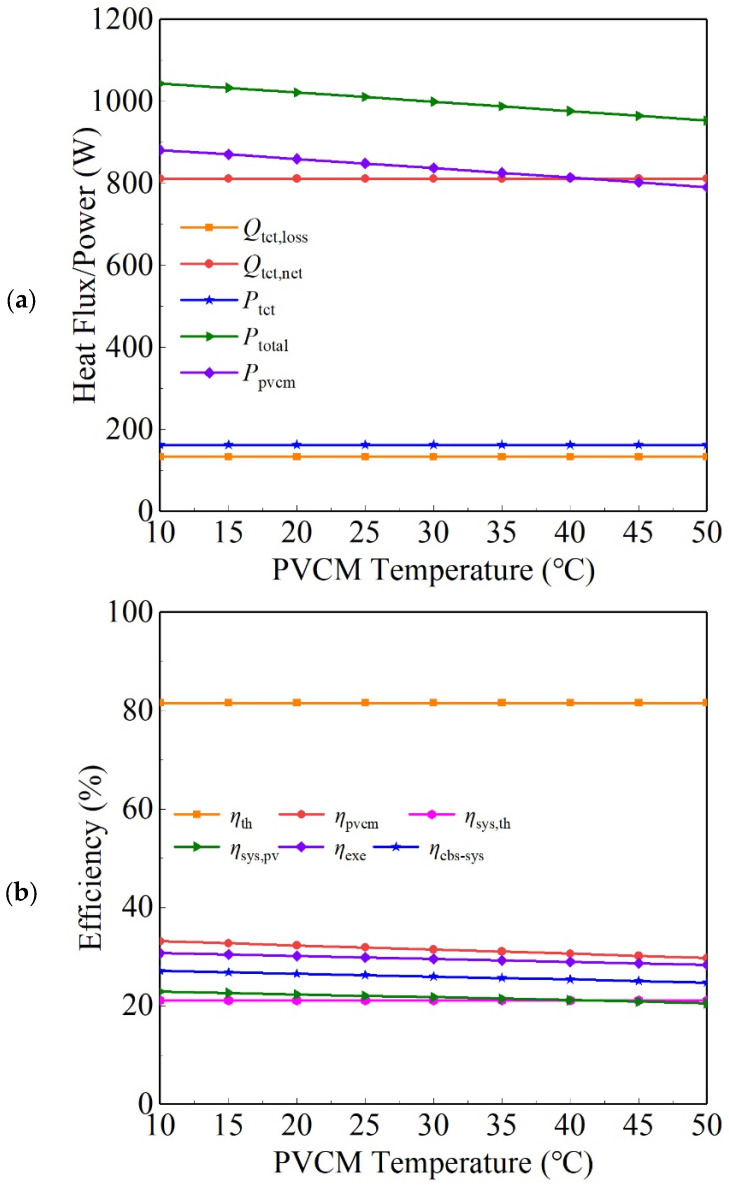
Powers (**a**) and efficiencies (**b**) of the CBS-PVT system when the PVCM temperature changes.

When *T*_tct_ is 200 °C and the solar irradiance is 1000 W/m^2^, with *T*_pvcm_ changed, the power and efficiency parameters of the CBS-PVT system are shown in Figure 8. The results reveal that when *T*_tct_ is fixed, with *T*_pvcm_ changed, *Q*_tct,loss_ of the TCT and *P*_th_ of the HUS both remain unchanged, but *P*_pvcm_ of the PVS decreases, and that results in a decrease in *P*_total_ of the CBS-PVT system. When *T*_pvcm_ rises from 10 °C to 50 °C, *P*_total_ decreases from 1043.5 W to 952.9 W.

According to Figure 8, when *T*_pvcm_ changes, *η*_th_ of the TCT and *η*_sys_,_th_ of the CBS-PVT system do not change. But, due to the reduction in *η*_pvcm_ of the PVS, *η*_sys,pv_, *η*_exe_, and *η*_cbs-pvt_ of the CBS-PVT system all decrease slightly. When *T*_pvcm_ rises to 50 °C, *η*_sys,pv_ and *η*_cbs-pvt_ are reduced to 0.205 and 0.248, respectively. Hence, to increase the system output power and energy efficiency, reducing the PVCM temperature and keeping it at a reasonably low level would be useful.

## 4. Conclusions

Solar PV–thermal technology is a cutting-edge technology in the field of solar energy utilization research. To increase the overall efficiency of PV utilization, a solar CBS-PVT system based on HTC and FSF is proposed and studied in this study. The approach of designing the CBS-PVT system is presented and the FSF used for this system is also designed, of which the average reflectivity and transmission rate are 0.272 and 0.728 for the full spectrum range. Performance investigation of the CBS-PVT system demonstrates that the optical processes can match the operating principle of the CBS-PVT system, which reveals the design correctness of the system. When the N-S STE is increased to 0.15°, the optical efficiency of the CSB-PVT system can be kept at 0.8653, which shows good adaptable capacity to the STE. The operation feasibility analysis demonstrates that the photoelectric efficiency of the PVS is 0.314, and the overall system efficiency is 0.26. Parametric analysis indicates that with an increase in the TCT operating temperature, the total output power and overall efficiency of the CSB-PVT system both rise first and then decrease. When the TCT temperature is about 225 °C, the CBS-PVT system reaches its maximum output power of 1003.6 W and the maximum overall efficiency of 0.261. When the PVCM temperature increases, the photoelectric efficiency of the PVS is reduced, resulting in linear decreases in the total output power and overall efficiency. When the PVCM temperature rises to 50 °C, the total power decreases to 952.9 W, and the photovoltaic and overall efficiencies of the entire CBS-PVT system decrease to 0.205 and 0.248.

The CBS-PVT system can have simpler layout and lower initial investment costs due to the usage of FSF, and compared with PV–thermal systems using parabolic trough or other large solar concentrators, the CBS-PVT system can have smaller field requirements as it employs the HTC. These make the CBS-PVT system suitable for applications in both large-scale and distributed PV–thermal utilization conditions.

## Figures and Tables

**Table 1:** Parameters of the CBS-PVT system.

Item	Value
HTC apertural diameter	800 mm
HTC focal distance	1600 mm
HTC length	300 mm
FSF height	1506.25 mm
FSF width	50 mm
PVCM height	1543.75 mm
PVCM width	30 mm
TCT diameter	30 mm
TCT height	1355 mm
Concentration ratio of the HTC	25.67
Relative aperture of the HTC	0.5

**Table 2:** The spectral filtering characteristics of the designed FSFs.

FSF Design Scheme	I	II	III	IV	V
Total layer number	5	9	13	17	25
$\rho_{\mathrm{ave}}$ (380~1100 nm)	0.059	0.065	0.105	0.082	0.095
$\tau_{\mathrm{ave}}$ (380~1100 nm)	0.941	0.935	0.895	0.918	0.905
$\rho_{\mathrm{ave}}$ (<380 nm, >1100 nm)	0.515	0.787	0.867	0.851	0.975
$\tau_{\mathrm{ave}}$ (<380 nm, >1100 nm)	0.485	0.213	0.133	0.149	0.025
$\rho_{\mathrm{ave}}$ (250~2500 nm)	0.164	0.232	0.279	0.258	0.272
$\tau_{\mathrm{ave}}$ (250~2500 nm)	0.836	0.768	0.721	0.742	0.728

**Table 3:** Constants in the thermodynamic estimation.

Item	Value	Item	Value
$V_{\mathrm{oc}}$	0.706 V	*T*_tct_	200 °C
$r_{s}$	0.012	*A*_tct_	0.471 m^2^
*A*_pvcm_	0.15 m^2^	ε	$2\times 10^{-7}T_{\mathrm{tct}}^{2}+5\times 10^{-5}T_{\mathrm{tct}}+0.05$ [27,28]
$\lambda_{2}$	1100 nm	$\eta_{\mathrm{plant}}$	0.9
$T_{0}$	25 °C	$\eta_{\mathrm{pb},\mathrm{net}}$	0.9
*T*_pvcm_	30 °C	$n_{f}$	1.28

**Table 4:** Thermodynamic estimation results.

Item	Result	Item	Result
*Q*_htc,in_	3850 W	$P_{\mathrm{total}}$	999.5 W
*Q*_pvcm,bs_	2662.7 W	$\eta_{\mathrm{pv}}$	0.314
*Q*_tct_	945.1 W	$\eta_{\mathrm{th}}$	0.816
*P*_pvcm_	837.4 W	$\eta_{\mathrm{sys},\mathrm{th}}$	0.211
*P*_tct_	162.1 W	*η*_cbs-pvt_	0.260

**Table 5:** Comparison of several PV–thermal systems.

PV–Thermal System	Concentrator	SF	$\eta_{\mathrm{sys},\mathrm{pv}}$	$\eta_{\mathrm{sys},\mathrm{th}}$
This study	HTC	FSF	0.218	0.211
Ref. [14]	Fresnel lens	Ag/CoSO_4_-PG	0.0764	0.46
Ref. [18]	Parabolic trough	ITO-Duratherm S	0.04	0.61
Ref. [29]	Fresnel lens	ZnO-EG	0.145	0.074

## Data Availability

The data presented in this study are available on request from the corresponding author.

## Nomenclature

*A*_PVCM_	total area of solar cell (m^2^)
*c*	velocity of light in the vacuum (m/s)
*CR*	concentration ratio (-)
*D*_t_	diameter of the TCT (mm)
*e*	charge of an electron (C)
*E*_g_	bandgap energy of the solar cells (eV)
*F*	focal length (mm)
*FF*	fill factor (-)
*h*	Planck constant (J·s)
*H*_f_	installation height of FSF (mm)
*H*_s_	installation height of solar cell (mm)
*H*_t_	installation height of TCT (mm)
*I*_sc_	short circuit current (A)
*k*_B_	Boltzmann constant (m^2^kg/(s^2^∙K))
*n_f_*	diode ideality factor of solar cell (-)
*P*_pvcm_	maximum power of the PV cell (W)
*P*_th_	output power of the HUS (W)
*P*_total_	total output power (W)
*QE*	external quantum efficiency (-)
*Q*_htc,in_	incident solar energy flux on the HTC (W)
*Q*_pvcm,bs_	solar radiation flux delivered to the PVCM (W)
*Q*_tct_	solar radiation energy reflected to the TCT (W)
*Q*_tct,loss_	solar radiation energy loss of the TCT (W)
*Q*_tct,net_	net heat flux obtained by the TCT (W)
*R*_a_	relative aperture (-)
*R*_g_	concentration ratio of the HTC (-)
*R*_t_	radius of the TCT (mm)
*T*_0_	environment temperature (°C)
*T*_pvcm_	temperature of solar cell (°C)
*T*_tct_	temperature of TCT (°C)
*V*_oc_	open circuit voltage (V)
*W*	width of the HTC (mm)
*W*_f_	width of the FSF (mm)
*W*_s_	width of the solar cell (mm)
*Z*	focal depth (mm)
Greek Symbols	
*ρ*	reflectance (-)
*τ*	transmittance (-)
*ε*_tr_	solar tracking error (deg)
*η*	efficiency (-)
*λ*	wavelength (nm)
Subscripts	
ave	average
bs	beam splitting
c	solar concentration
cbs-pvt	CBS-PVT system
exe	exergic
fsf	film spectrum filter
htc	half-trough concentrator
opt	optical
pv	PV
pvcm	PV cell module
sys	system
tct	thermal collector tube
th	thermal
tr	tracking
Abbreviations	
CBS-PVT	concentrating beam splitting photovoltaic thermal
C-PV	concentrating photovoltaic thermal
EFD	energy flux density
EG	ethylene glycol
HTC	half-trough concentrator
HTF	heat transfer fluid
HUS	heat utilization subsystem
ITO	indium tin oxide
FSF	film spectral filter
NSF	nanofluid spectral filter
PDMS	polydimethylsiloxane
PG	propylene glycol
PV	photovoltaic
PV–T	PV and thermal
PVCM	PV cell module
PVS	photovoltaic subsystem
SF	spectral filter
STE	solar tracking error
TCT	thermal collector tube
